# Flowers, Ferns, and Ward Decorations

**Published:** 1887-04-02

**Authors:** M. Mattocks


### Flowers, Ferns, and Ward Decorations.

[Continued from page 298.—Communications for this column should be addressed "Gardener," care of the Editor of The Hospital.]

In the useful hints which have appeared in The Hospital on ward decoration, I have noticed the omission of one point, namely, that a hospital ward may be most effectively ornamented from the *outside*. Wherever there is a window-sill wide enough to put a box upon, a show may be kept up for nine months of the year with flowers that would speedily wither if kept *inside*. Except the windows open with casements, there is nothing to prevent the sills being utilised in this manner, and there ought to be no difficulty in providing the boxes. Many a carpenter or joiner must remember the hospital ward in which he or one of his family has received all the aid that human skill could give in alleviation of sickness or accident, and must wish to show his gratitude if he knew how. For such a man to make a box to fit any window-sill would cost very little time or money. The fronts can be made pretty in many ways, with ornamental painting, coloured tiles, sea or snail-shells (stuck in a layer of Portland cement, which can be made to adhere by the box being stuck plentifully with small tacks), pieces of virgin cork-rustic sticks (fastened on with long fine nails), and other modes; nor need there be any want of suitable plants to fill them. For climbing at the sides of the window nothing can exceed the ivy in its evergreen hardihood, to which in summer can be added the gay and varied nasturtiums, sweet-peas, canary creeper, and in sunny places, convolvulus major, and if the box is deep, clematis. For the middle, anything which the air of the place will allow to grow in beds, will do equally well in window-boxes, with very little care. It may be said that nurses have not the time, nor all of them the skill to become window-gardeners. I see no reason why they need. Large hospitals in their own grounds have usually a man to keep them in order, who could fill and plant the boxes from time to time, leaving only the watering to be done from the wards, and in other places it would be very easy for ladies and gentlemen, who have gardens and gardeners, each to provide for one ward. The competition between them would surely give far more real pleasure than the most coveted prizes at flower-shows! In this, as in other things,

"The thoughtful love,
Through constant watching, wise,"

would give better results than the mere outlay of money. M. Mattocks.

### AN INVALUABLE AND NECESSARY INSTITUTION.

A hospital is an invaluable and necessary institution, where the sick and wounded are well cared for, doctored, and nursed. Many a poor sufferer has been able to pass his last moments on this earth, in peace and comfort in one of these homely institutions; but for them, in misery. —Perseverance.

